# Development, Optimization and Characterization of Eudraguard^®^-Based Microparticles for Colon Delivery

**DOI:** 10.3390/ph13060131

**Published:** 2020-06-24

**Authors:** Claudia Curcio, Antonio S. Greco, Salvatore Rizzo, Lorena Saitta, Teresa Musumeci, Barbara Ruozi, Rosario Pignatello

**Affiliations:** 1 Department of Drug Sciences, Section of Pharmaceutical Technology, University of Catania, 95125 Catania, Italy; clacurcio@yahoo.it (C.C.); antonio87251@gmail.com (A.S.G.); whitelight@live.it (S.R.); teresa.musumeci@unict.it (T.M.); 2 Department of Civil Engineering and Architecture (DICAR), University of Catania, 95125 Catania, Italy; lorena.saitta@phd.unict.it; 3 Department of Life Sciences, University of Modena and Reggio Emilia, 41100 Modena, Italy; barbara.ruozi@unimore.it

**Keywords:** food-grade acrylate polymers, quercetin, DSC, FT-IR, PXRD, enteric release, drug delivery

## Abstract

Development of pH-dependent systems for colon delivery of natural active ingredients is an attractive area of research in the field of nutraceutical products. This study was focused on Eudraguard^®^ resins, that are methacrylate copolymers approved as “food grade” by European Commission and useful for the production of food supplements. In particular, Eudraguard^®^ Biotic (EUG-B), characterized by a pH-dependent solubility and Eudraguard^®^ Control (EUG-C), whose chemical properties support a prolonged release of the encapsulated compounds, were tested. To obtain EUG microparticles, different preparation techniques were tested, in order to optimize the preparation method and observe the effect upon drug encapsulation and specific colonic release. Unloaded microparticles were initially produced to evaluate the influence of polymer characteristics on the formulation process; subsequently microparticles loaded with quercetin (QUE) as a low solubility model drug were prepared. The characterization of microparticles in the solid-state (FT-IR spectroscopy, differential scanning calorimetry and X-ray diffractometry) indicated that QUE was uniformly dispersed in a non-crystalline state in the polymeric network, without strong signs of chemical interactions. Finally, to assess the ability of EUG-C and EUG-B to control the drug release in the gastric environment, and to allow an increased release at a colonic level, suitable in vitro release tests were carried out by simulating the pH variations along the gastro-intestinal tract. Among the evaluated preparation methods, those in which an aqueous phase was not present, and in particular the emulsion-solvent evaporation method produced the best microparticle systems. The in vitro tests showed a limited drug release at a gastric level and a good specific colon release.

## 1. Introduction

Targeted ileo-colon delivery is a highly desirable approach for the local treatment of a variety of bowel diseases such as ulcerative colitis, Crohn’s disease, colon cancer. Additionally, the selective release in this area of sensitive compounds such as proteins and peptides can improve their systemic bioavailability.

Colon-specific drug delivery systems (CDDS) should be capable of protecting the drug in the stomach and in the small intestine allowing drug release and absorption once the system reaches the colon [1]. CDDS can possess several advantages, including reduction of drug payload and of the incidence of side effects, bypassing the liver first pass metabolism, improving patient compliance, e.g., in terms of frequency of administration, reducing the gastric irritation caused by some drugs, and improving the local bioavailability of poorly absorbed drug molecules [2].

Planning a CDDS introduces various challenges and restrictions, since a colon-targeted oral dosage must cross the entire gastro-intestinal canal (GIT) to reach the final site of action. The complex physiology of GIT can render this a problem, as it presents different pH values, fluid volumes and transit times, highly variable at an intra- and inter-individual level. The presence of food and metabolic enzymes also increase GIT physiological complexity [3].

Conventional oral dosage forms are almost ineffective in delivering high amounts of drugs in the colon, due to the absorption and/or degradation of the active ingredient in the upper sections of GIT. Various mechanisms have been proposed to target oral drugs to the colon: they include pH-dependent systems, time-dependent release systems, microbially triggered DDS, and osmotically controlled CDDS [1,4,5].

Currently, the choice of a DDS is increasingly oriented towards the micro- and nanometric vectors, which in terms of colon delivery, and in particular in the treatment of inflammatory bowel disease (IBD) can exert positive features such as an increase of residence time in the colon [6,7], selective release in the inflamed tissue, absorption by the immune cells, present in large numbers in the inflamed regions of colon [8], reduction of the dosage form elimination through diarrhea, a common symptom in IBD [9].

In particular, microparticles showed a tendency to mucoadhesion onto the wall of the intestinal mucosa [10], with a limited absorption through the epithelial barrier. An accumulation of microparticles in the rectal mucosa of patients has also been observed, while nanoparticles were detectable only in traces. Conversely, nanoparticles can pass into the serum compartment, producing systemic effects [11].

This study focused on the pH-dependent drug release strategy. It is based upon the change of pH along the different parts of GIT, from the acid value of the stomach (around 1–2, and up to 4 after eating), to the neutral small intestine (pH 6–7), up to a value around 7–7.5 in the distal ileum. Coating with pH-sensitive polymers tablets, capsules or pellets ensures to withstand the stomach and the proximal part of the small intestine and then release the drug at the neutral or slightly alkaline pH level of the terminal ileum and colon. This ultimately ensures a delayed release and protect the active compound from the gastric fluid, if necessary.

To achieve this type of release, various polymers, such as pectin, hydroxypropyl- methylcellulose phthalate (HPMC-P), HPMC acetate succinate (HPMC-AS), cellulose acetate phthalate (CAP), cellulose acetate trimellilate (CAT), and polyvinyl acetate phthalate have been explored [12,13,14]. Pharma industry however mainly directs its attention to poly(methacrylic acid-co-methyl methacrylate) resins (Eudragit^®^ L, S and F) as coating polymers of solid oral dosage forms.

In order to investigate the possibility of producing microparticle systems for the colonic release of natural active ingredients, we drove our attention to Eudraguard^®^ resins. Eudraguard^®^ products have been approved as food-grade ingredients and are suggested as coating materials for solid oral dosages in the nutraceutical field to prolong the release of actives, to give gastric resistance, to mask unpleasant odour and tastes and to protect against moisture [15].

Among the four types of commercialized resins, Eudraguard^®^ Biotic (E 1207) (EUG-B) and Eudraguard^®^ Control (E 1206) (EUG-C) have been selected for this research. EUG-B (poly(methyl acrylate-co-methyl methacrylate-co-methacrylic acid)) is a fully polymerized anionic copolymer of methyl acrylate, methyl methacrylate and methacrylic acid, in a 7:3:1 ratio respectively. The ratio of the free carboxyl groups to the ester groups is approximately 1:10. EUG-B has been approved by the European Commission as a safe food additive and is employed in the enteric/delayed-release coating to permit the pH-dependent release of nutrients or active ingredients from oral dosage forms, in particular at colonic level, since it becomes soluble in water only above pH 7.0 [15,16].

EUG-C (poly(ethylacrylate-co-methyl methacrylate)) is a fully polymerized neutral copolymer of methyl methacrylate and ethyl acrylate in a 2:1 ratio. This polymer allows the continuous release of substances which are thus more easily absorbed for a prolonged and defined period of time [15]. It has been approved by EFSA as a “food additive” and since January 2014 it has been self-established as a GRAS material, as confirmed also by various toxicity studies [17].

Recently, EUG-B has been investigated as an enteric coating material of non-dairy probiotics with the main aim of protecting bacteria from degradation in the gastrointestinal tract and obtain a selective release at gut level [18].

The physico-chemical characteristics of these polymers have attracted our attention towards the possibility of using them, instead than as coating polymers, to produce microparticulate systems for a colon targeting strategy of natural active compounds. An analogous strategy has been largely exploited by some of us with the corresponding pharma-grade resins, Eudragit^®^ RL 100 and RS 100 [19,20,21].

3,3′,4′,5,7-Pentahydroxyflavone (QUE) is natural flavonoid and the major representative of flavonol subclass. Scientific research proved that dietary polyphenols possess protective and therapeutic effects in the management of IBD mediated via down-regulation of inflammatory cytokines and enzymes, enhancing antioxidant defense, and suppressing inflammatory pathways and their cellular signaling mechanisms [22]. In vivo evidences showed that dietary QUE could ameliorate experimental colitis in part by modulating the anti-inflammatory effects and bactericidal capacity of macrophages through a heme oxygenase-1 (HO-1)-dependent pathway [23]. It has been suggested that QUE can restore the right intestinal host-microbioma association by rebalancing the pro-inflammatory, anti-inflammatory and bactericidal capacity of gut macrophages [24,25].

Poor stomach and intestinal absorption of flavonoids are the main issue that limit the concentration reached in the colon [26]. Therefore, CDDS have been studied in the last years as a strategy to face these drawbacks. For instance, chitosan/nutriose-coated vesicles showed to improve QUE concentration in the colon [27]. Guazelli et al. [28] reported that QUE-loaded microcapsules were more effective in pharmacological animal models of colitis compared to neat QUE. Similarly, other natural or synthetic polymeric materials have been explored to achieve a pH-controlled oral release of QUE and to improve its chemical and enzymatic stability [29,30].

This work was focused towards the operative and formulative optimization of Eudraguard^®^-based microparticle systems, investigating different preparation techniques: Quasi-emulsion Solvent Diffusion (QESD), Solvent Evaporation (SE); co-evaporation (CoE), solvent casting (SC), and Emulsion-Solvent Evaporation (ESE). Quercetin (QUE) was encapsulated in microparticles as a model active compound with poor aqueous solubility.

The produced EUG microparticles were characterized in the solid state and the pH-controlled release of model compounds was assessed at the various pH values of GIT.

## 2. Results and Discussion

### 2.1. Assessment of Microparticle Preparation Methods

Comparing the different preparation techniques used, the main information emerging is that the presence of an aqueous phase in the production process does not lead to a homogeneous microparticle population. For instance, during the homogenization step of the QESD technique the formation of rubbery filaments and masses occurred, caused by the irregular aggregation of the polymer upon contact with water. An increase of surfactant concentration also did not allow one to solve the hitch (data not shown). Upon removal of such aggregates, analysis of the suspension by dynamic light scattering (DLS) showed the presence of homogenous populations of nanoparticles, with a mean size ranging from 77 to 165 nm, with a polydispersity index always below 0.2 (data not shown). Although these systems could deserve attention for a potential application in drug delivery, they were out of scope of the present study, and thus this preparation method was not further investigated.

With the SE technique the formation of large polymeric clusters also occurred, due to the interactions at the acetone-water interface. Therefore, anhydrous formulation approaches were investigated in the sequel of the study. In particular, the techniques of solvent casting (SC) and emulsion-solvent evaporation (ESE) appeared to be the more suitable ones to produce the wished microparticles.

Homogeneous, thin and easily foldable elastic films with a rubbery consistency were obtained using the SC technique. The advantage of this technique consists in the formation of a polymeric film with a homogeneous thickness. The solubility of the polymer and the drug in the chosen common solvent plays a fundamental role in this process: if the precipitation of the two components occurs at very different times, the polymeric network formed is unable to efficaciously incorporate the drug, which precipitates separately; conversely if the difference in solubility (and precipitation time) between the two ingredients is low, the polymer matrix is able to incorporate the drug. Finally, if a co-precipitation occurs, e.g., both ingredients precipitate at the same time (ideal condition), the polymer incorporates homogeneously the drug into its forming network.

At this stage of the study, these films were not studied in deep, apart from characterization and drug release tests, but it is predictable that they could represent interesting structures to produce innovative controlled release formulations of drugs and bioactive compounds at an intestinal level.

**Figure 1:**
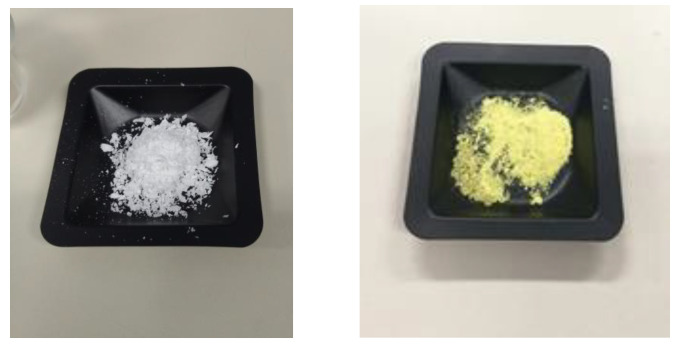
Appearance of blank (**left**) and QUE-loaded EUG-B microparticles (at a 1:10 drug-to-polymer ratio) (**right**) produced by the ESE technique.

The ESE technique allowed the production of homogenous powders using 100% EUG-B (Figure 1) but gave gummy flakes with 100% EUG-C polymers. A test was carried out using a 1:1 ratio between the two polymers, resulting is a heterogeneous powder, with some aggregates that were easily powdered with a mortar.

Furthermore, systems with different drug-to-polymer ratios were tested to identify the best concentration of polymer able to form an optimal system for colonic release. In particular, the evaluation concerned the EUG-B polymer, with drug-polymer ratios of 1: 5, 1:10 and 1:25 with quercetin (QUE) as a model compound. It has been observed that as the concentration of polymer increased, the obtained systems had a progressively less intense yellow color and were less smooth, due to the increase in cohesive forces during solvent evaporation.

The CoE method aimed at evaluating the formation of discrete microparticles upon removal of the solvent by rotary evaporation in vacuo. In the first attempts, rubbery films difficult to collect and grind were obtained; for this reason, it was chosen to add a solid excipient as anti-caking materials to facilitate the film formation and recovery process. Some excipients commonly used as disintegrants or glidants in the production of solid oral pharmaceutical forms, such as talc, cellulose derivatives and silicates have been tested.

Methylcellulose was excluded after the first tests as it did not allow to obtain a powder with the polymer at any weight ratio considered. Preparations with colloidal silica immediately gave a powder material, while in the case of the other excipients, at higher polymer-to-excipient ratios the formulations had to be worked with a mortar to obtain finer and more homogeneous powders.

### 2.2. Morphological Characterization of Microparticles by SEM

SEM analysis was made to determine mean sizes of the various ESE microparticle batches and to study their surface morphology. Very interesting results were observed by the comparison of the systems produced with increasing QUE−EUG-B weight ratios (cf. Table 1). First of all, none of the batches produced by this method showed signs of large particle aggregation.

**Figure 2:**
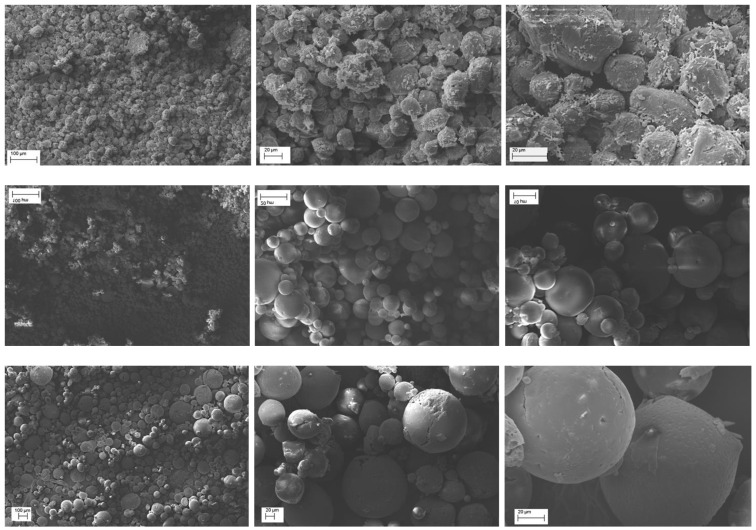
SEM pictures of ESE.B.Q5 (**top**), ESE.B.Q10 (**middle**), and ESE.B.Q25 microparticle batches (**bottom**) at various magnification.

An immediate evidence was that at a 1:5 ratio (Figure 2, top), whose mean particle size was in the range 15-25 µm, the drug remained for a large part onto the surface of microparticles. Conversely, at 1:10 and 1:25 drug-polymer ratios the surface pf microparticles was smooth and no trace of free drug powder was visible (Figure 2). The meaning of these findings was that a minimum ten-fold amount of EUG-B was necessary for a complete encapsulation of QUE. The size of the microparticles ranged between 10 and 30 µm with the 1:10 ratio, and between 50 and 130 µm for the 1:25 ratio.

Only in the ESE.B.Q25 microparticles the highest magnification revealed some small pores/holes on their surface; moreover, the internal structure of the microparticles appears to be uniform and compact (see Figure 2, bottom).

The very attractive morphology of these microparticles confirms the capacity of the ESE technique to generate smooth and uniform micro/nanostructures when a solution of the polymer in acetone or ethanol is emulsified with paraffin oil and then precipitated by addition of a non-solvent mean. This technique was already successfully used by some us with Eudragit^®^ Retard copolymers [31] and was thereafter left aside because of the discomfort of removing the residual mineral oil through washings with highly volatile solvents. However, the outcomes of our experiments seem to confirm the efficacy and suitability of this method for the production of acrylate-based microspheres.

### 2.3. Solid-State Analysis of ESE Microparticles

To highlight the interaction between QUE and the polymer matrix in the preparations obtained with the ESE technique, FT-IR analysis was performed. Figure S1 shows the IR spectrum of pure QUE, whose explanation is given in Table 2.

Some exemplificative FT-IR spectra of EUG-B and EUG-C, blank and QUE-loaded ESE microparticles (1:10 drug-to-polymer ratio) and a corresponding physical mixture (1:10 QUE-EUG-B) are reported in Figures S2–S9. 

The IR spectra of blank EUG-C and EUG-B+C ESE microparticles did not show relevant differences compared to the pure (dried) starting copolymers, as expected considering that the ESE production method did not expose the material to high temperatures and aggressive solvents. In the IR spectra of microparticles and QUE−EUG-B 1:10 physical mixture various signals were registered in the fingerprint region between 500 and 1600 cm^−1^, with a reduced intensity than the corresponding spectra of neat QUE and polymer. Such a behavior endorses that QUE was dispersed in an intact form in the polymeric matrix, as already shown in other studies [33]. 

In fact, overlapping these spectra no relevant shift in the resonance frequencies was noticed, with respect to pure QUE and copolymer (EUG-B), which instead would have suggested a strong modification of the atomic and electrostatic surrounding. The last hypothesis was further supported by the absence of relevant changes in the region of OH stretching band (3200–3600 cm^−1^) and in that one of C=O stretching (around 1735 cm^−1^), whose modification in position and intensity would have indicated a strong chemical interaction between the carbonyl and the carboxyl groups of the flavonoid and the polymer [34].

To confirm the IR data, DSC and PXRD analyses were carried out. In both studies, QUE, EUG-B, ESE formulations and the corresponding physical mixtures (equivalent to each microparticle compositions) were assayed for comparison.

**Figure 3:**
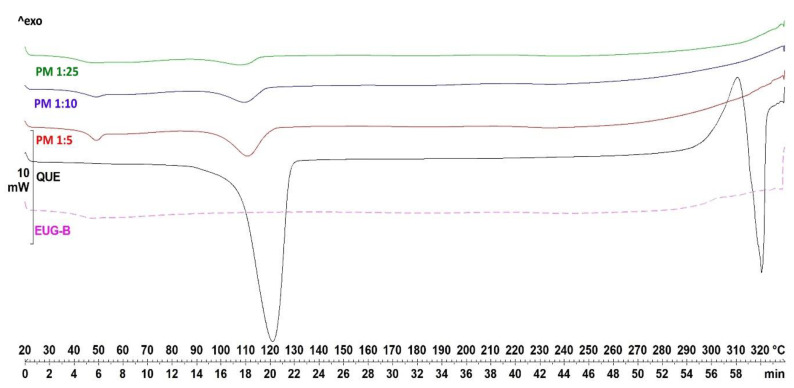
DSC curves of neat QUE, dried EUG-B and QUE−EUG-B physical mixtures.

DSC analysis was performed to assess the degree of crystallinity or amorphization of QUE dispersed in the polymer matrices. The calorimetric curve of pure QUE showed a strong endothermic peak at 320 °C, which corresponds to the melting point and which indicates its crystalline nature (Figure 3). A first strong endothermic peak is also present around 120 °C, related to the water loss of the sample, as already confirmed by thermogravimetry (TG) and photovisual analysis [35].

The DSC thermogram of dried EUG-B (Figure 3) showed a weak endothermic peak at 48 °C attributable to the glass transition of the amorphous polymer (T*_g_*). When blank ESE and CoE microparticles were submitted to DSC, thermograms similar to those of the pure polymer were obtained (not shown), indicating that the polymers did not undergo any physico-chemical change during the dissolution and drying processes. Analyzing the physical mixtures of QUE and EUG-B (Figure 3), at the same weight ratios used in the microparticles, it was possible to observe small peaks around 100–120 °C, whose intensity was proportional to the amount of drug in the mixture, corresponding to the dehydration of QUE observed for the neat drug. Even if the production of these physical mixtures was realized without addition of any solvent, the absence of the melting peak at higher temperature suggested that some kinds of interactions occurred between the drug and polymer, which affected the crystalline structure of QUE.

**Figure 4:**
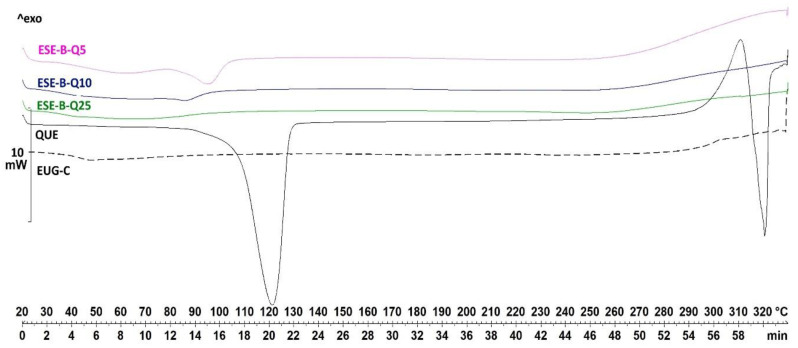
DSC curves of EUG-B ESE microparticles loaded with different amounts of QUE (see Table 1 for microparticle composition).

The DSC curves of ESE microparticle produced with increasing QUE-polymer weight ratios (Figure 4) showed that, after the microencapsulation, no thermal transition corresponding to pure QUE was visible, while the dehydration peak at lower temperature was strongly reduced in intensity; only the system produced with a higher amount of EUG-B (ESE.B.Q25) showed a small shifted peak that corresponds to the T*_g_* of the polymer.

**Figure 5:**
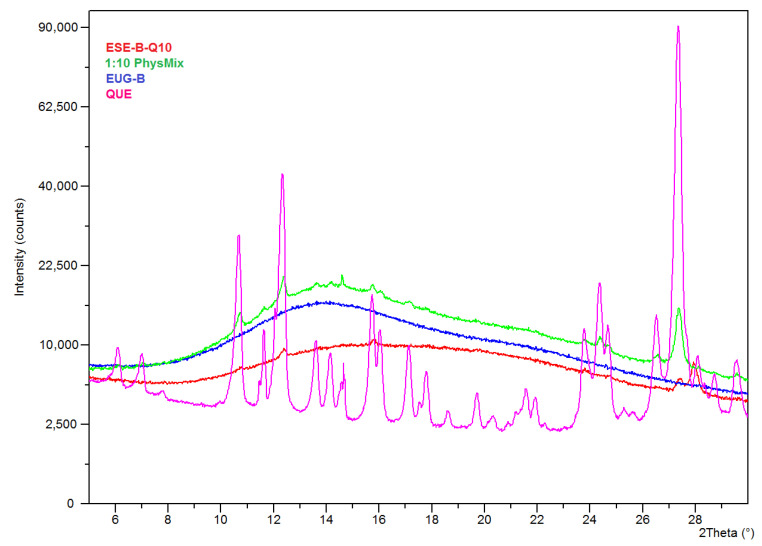
PXRD spectra of neat QUE, dry EUG-B and 1:10 QUE-loaded ESE microparticles and the corresponding physical mixture.

These findings indicate that the encapsulated QUE is present in a noncrystalline or highly microcrystalline disordered state within the polymeric network. To support such a hypothesis, PXRD analyses were carried out. The X-ray diffractograms showed that the characteristic peaks of neat QUE almost completely disappeared in the spectrum of the EUG-B ESE microparticles obtained at a drug-polymer ratio of 1:10 (Figure 5). This highlights that QUE was homogeneously dispersed within the polymeric matrix, most probably as a molecular dispersion or solid solution, as already observed in previous similar studies with Eudragit^®^ copolymers [14,36]. On the other hand, such observation perfectly complies with what observed by the SEM analysis of this batch (Figure 2).

**Figure 6:**
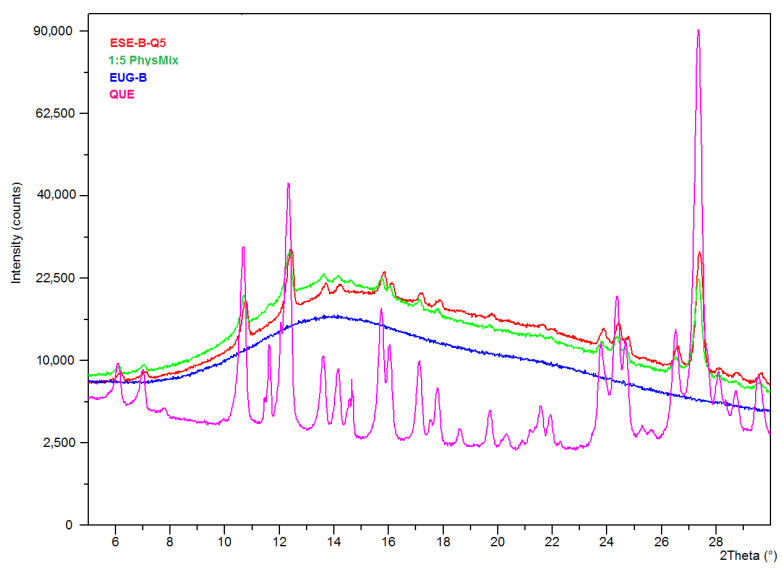
PXRD spectra of neat QUE, dry EUG-B and 1:5 QUE-loaded ESE microparticles and the corresponding physical mixture.

Conversely, the corresponding diffractogram of the microparticles prepared at a 1:5 drug-polymer weight ratio (Figure 6) showed the proper peaks of QUE, in a similar manner than in the corresponding physical mixture. This suggested an incomplete encapsulation of the drug, with a fraction that remained located on the external surface of the microparticles. Also in this case, PXRD data are perfectly in line with the information suggested by the that SEM analysis (Figure 2). Morever, the absence of shifts of the peak position in these diffractograms further supported the conclusion that QUE did not develop significant chemical interactions with the EUG matrix.

### 2.4. Drug Content and Encapsulation Efficiency Evaluation

The microparticles produced by CoE gave EE values close or below 50%, suggesting a relevant loss of drug during the preparation steps, linked to the extraction and grinding processes (Table 3).

ESE samples (Table 4) gave much higher values, indicating that this method allows an almost quantitative recovery of the drug after the production steps.

### 2.5. In Vitro pH-Dependent Drug Dissolution and Drug Release Patterns

**Figure 7:**
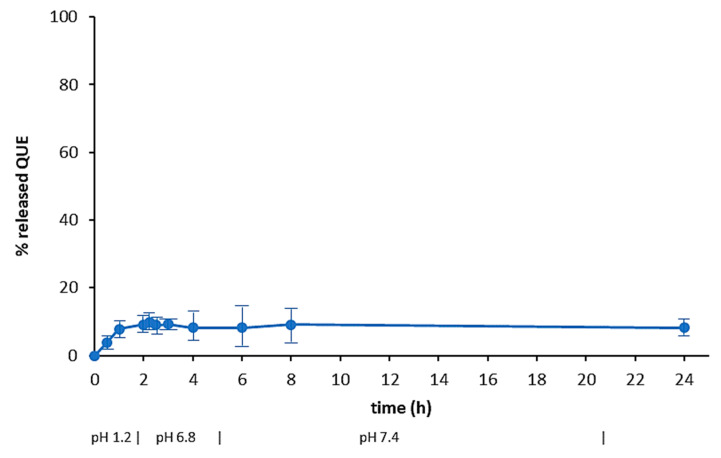
Dissolution curve of QUE at 37 °C in aqueous media with pH changes (0–2 h: pH 1.2; 2–6 h: pH 6.8; 6–24 h: pH 7.4) (mean of 3 experiments ± s.e.)

To determine the dissolution rate of the neat drug, as well as its release from microparticles, as a function of pH, samples were subjected to a drug dissolution test (paddle method) by reproducing the physiological conditions of the gastrointestinal tract. The different conditions of the GI tract were mimicked using simulated gastric fluid (pH 1.2) for 2 h, then simulated intestinal fluid (pH 6.8) for 4 h and finally a phosphate buffered solution at pH 7.4 up to 24 h. The dissolution profile of neat QUE is shown in Figure 7, that confirmed the poor solubility of the drug in aqueous media.

The pH-dependent release test allows to evaluate the ability of a polymer to limit and/or control the release of the entrapped drug in the gastric environment, and to obtain a prolonged ileo-colonic release. This assay was applied to CoE, SC and ESE systems, that produced the more suitable dry microparticles.

Yus et al. have recently proven by a microscopic examination the behavior of EUG-B microparticles at the various pH values of the GIT [20]. The polymer remained insoluble both in gastric and enteric environments but, while at acidic pH the microparticle did not show relevant signs of degradation, a progressive surface erosion started at the intestinal pH, with the formation of a porous structure and the consequent release of the entrapped cargo; therefore, a control of QUE release as a function of pH value was presumable in the tested microparticles.

**Figure 8:**
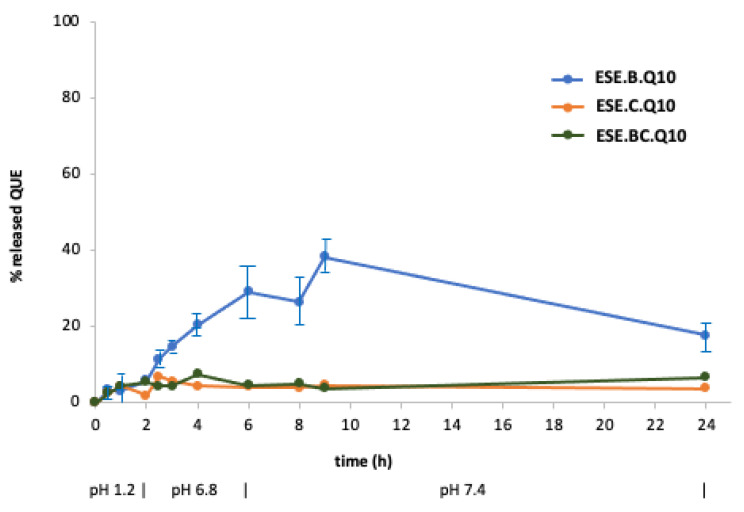
Release profile at 37 °C of QUE from microparticles made by the ESE technique (at 1:10 drug-to-polymer weight ratio) in aqueous media with pH changes (0–2 h: pH 1.2; 2–6 h: pH 6.8; 6–24 h: pH 7.4) (mean of 2 or 3 experiments ± s.e.; s.e. values batches ESE.C.Q and ESE.BC.Q were all within ±3%).

Figure 8 displays the release profiles of the formulations obtained by ESE with different ratios between the two polymers (Table 1). The pattern of ESE.B.Q10 (100% EUG-B; 1:10 drug-to-polymer ratio), indicated a reduction in the gastric release of QUE, a maximum value of 5%, and a gradual intestinal release, with a peak of 28% at pH 6.8 and up to 40% in the colonic environment. The profile of ESE.C.Q10 (100% EUG-C) confirmed the typical release behavior of this polymer, with a maximum 5% release at acidic pH and the achievement of a plateau after 4 h in the remaining portions of the gastrointestinal tract. The ESE.BC.Q10 formulation, produced using a 1:1 ratio between the two polymers, showed a release profile similar to the previous one: this confirms that the presence of EUG-C polymer did not positively contribute to control a pH-dependent release of QUE.

Noteworthy, in these and following release tests a reduction of QUE concentration in the receiving medium was observed, although a correction made to balance the various sample withdraws. This trend has been measured also in the dissolution curve of neat QUE (Figure 7), although not easy to appreciate visually. A possible explanation is that this compound undergoes a progressive physico-chemical degradation in alkaline media, but already at pH > 7 at 37 °C, with, among others, a consequent reduction of UV peak intensity, as already reported by other authors [37]. Using an UV spectrophotometric determination of drug release, therefore, an apparent lowering of QUE concentration was observed.

**Figure 9:**
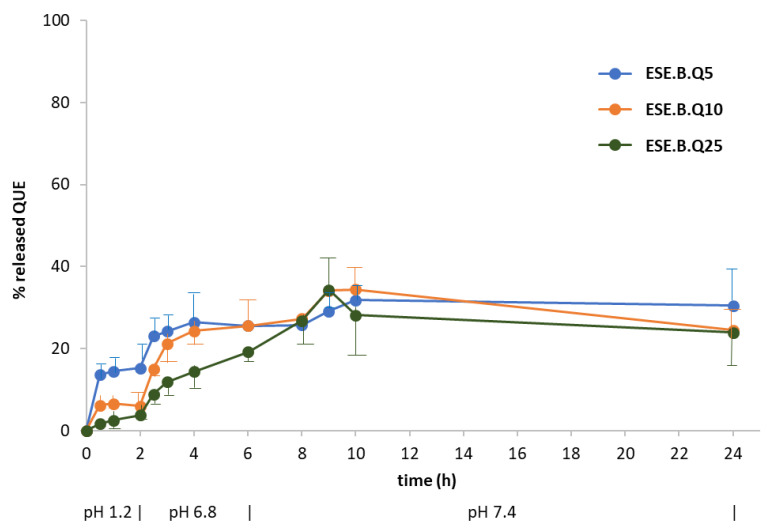
Release profile at 37 °C of QUE from EUG-B microparticles made by the ESE technique at different drug-to-polymer weight ratios, in aqueous media with pH changes (0–2 h: pH 1.2; 2–6 h: pH 6.8; 6–24 h: pH 7.4) (mean of 2 replicates ± s.e.). The composition of microparticles is given in Table 1.

Subsequently, the same release test was reiterated on the EUG-B formulations produced at different drug-to-polymer ratios (from 1:5 to 1:25; cf. Table 1). Comparing the respective drug release patterns (Figure 9), it was evident that increasing the relative amount of copolymer led to a reduction in gastric release, from 15% to 3% of the encapsulated QUE. Noteworthy, the behavior of the microparticles made at an 1:5 QUE−EUG-B ratio (ESE.B.Q5 batch) agreed with the microscopic evidence (Figure 2, top) and PXRD results (Figure 6) that a certain amount of drug remained onto the surface of microparticles, being released quickly within the first 30 min of the test at pH 1.2, a value where the polymer matrix is totally insoluble.

Likewise, at an intestinal level, a reduction of drug release was observed as a function of drug-to-polymer ratio (with a value around 10% after 6 h for the 1:25 batch). In the colon setting no noticeable difference between the various samples was detected, with an approximately 40% drug release, suggesting that at such pH conditions the permeability of the polymeric matrix to QUE reached a maximum value, independently from the relative drug-to-polymer amounts.

**Figure 10:**
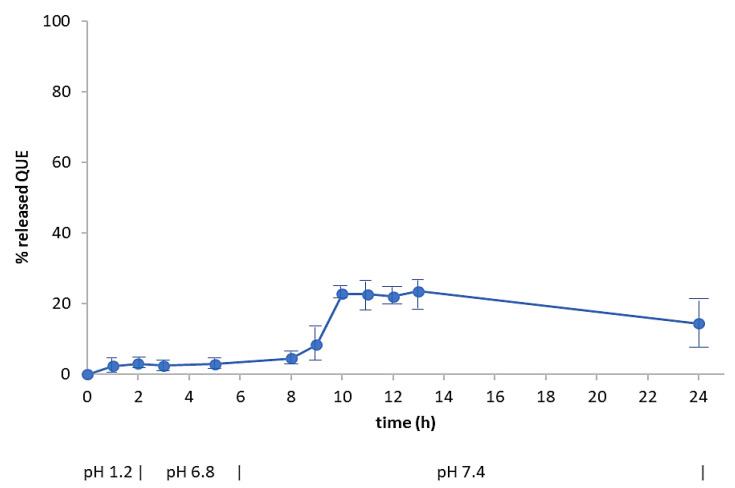
Release profile at 37 °C of QUE from microparticles made by the SC technique in aqueous media with pH changes (0–2 h: pH 1.2; 2–6 h: pH 6.8; 6–24 h: pH 7.4) (mean of 2 replicates ± s.e.).

The microparticles made by the SC technique displayed a profile characterized by the absence of gastric and intestinal release, with a localized release of QUE in the colonic tract (Figure 10).

It would then seem that the slow evaporation of the solvent from a co-solution between QUE and EUG-B led to a more homogeneous matrix, with a double positive effect: first, a reduced presence of free drug on the surface of microparticles, with a consequent limited loss of it in the initial phase of the release test. Secondly, a more effective capacity of the polymer to assure a pH-controlled drug release, since the diffusion of QUE occurred only above pH 7, i.e., when the polymer itself is soluble in water.

**Figure 11:**
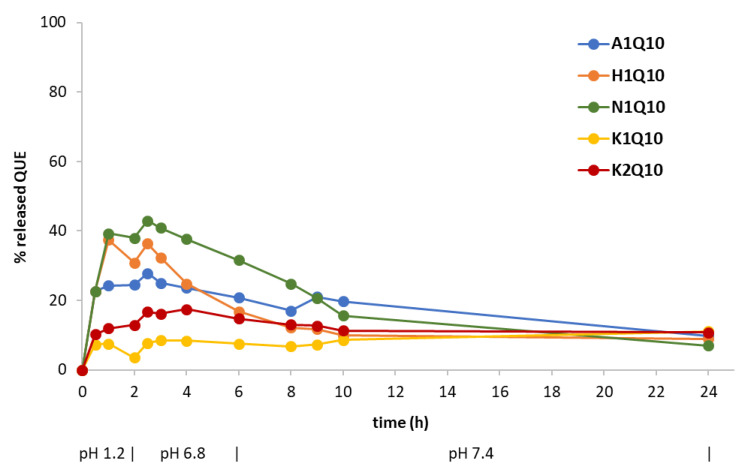
Release profile at 37 °C of QUE from microparticles made by the CoE technique in aqueous media with pH changes (0–2 h: pH 1.2; 2–6 h: pH 6.8; 6–24 h: pH 7.4). Values are the mean of two replicates; s.e. bars were not reported for the sake of clarity, but values were within ±5% (H1Q10) or within ±4% for the other batches.

By the CoE technique, QUE-loaded EUG-B microparticles at a 1:10 weight ratio were formulated. Furthermore, some pharmaceutical excipients were added, selected according to the macroscopic aspects of the respective empty systems (Table 5). All the formulations were subjected to the release test with the exception of two preparations added with Kollidon CL [K3Q10 (2:1) and K4Q10 (3:1)], as they were difficult to collect as powders from the evaporation vessel, probably because of the excess of polymer. Figure 11 indicates that no inhibition of gastric release of QUE was achieved using HPMC (H1Q10) and Neusilin^®^ (N1Q10), whereas by adding Aerosil^®^ (A1Q10) only a partial control of drug release in the acidic medium was accomplished. Again, the relevant amount of drug released within the first 2 h of the assay would suggest that, using this production technique, a large fraction of QUE was not incorporated in the polymeric network, but remained in the state of a physical mixture onto the surface of microparticles.

The absence of a further release of the drug in the intestinal medium could be explained with the consistency and scarce porosity of these systems. Addition of Kollidon CL (K1Q10 and K2Q10 batches) even further limited the release of the encapsulated drug at the different pH, further confirming the hardness of microparticles and the necessity of an optimized solid dosage form, e.g., by addition of some disgregants.

**Figure 12:**
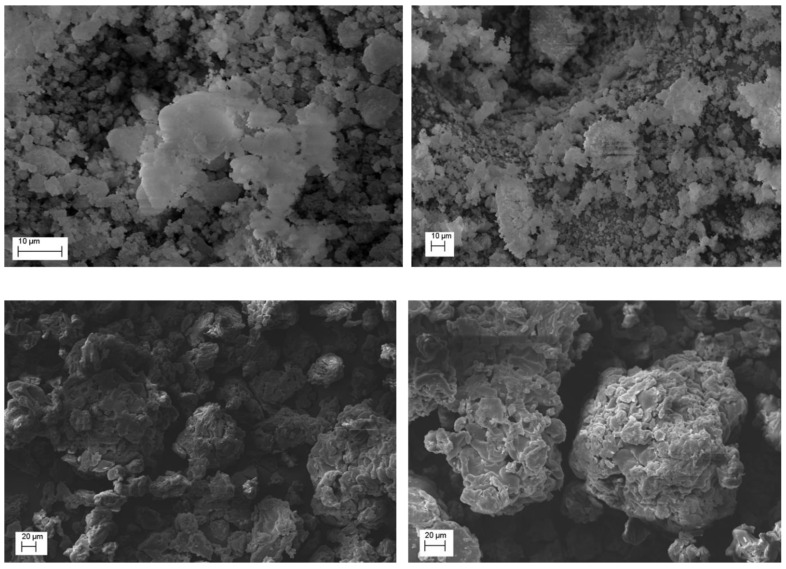
SEM pictures at various magnifications of N1Q10 (**top**) and K1Q10 (**bottom**) microparticle batches produced by the CoE method (see Table 5 for composition).

A corroboration to this hypothesis came from the SEM analysis of microparticles N1Q10 and K1Q10, i.e., two samples that showed very different drug release profiles in vitro (Figure 11). As Figure 12 shows, while the EUG-B CoE microparticles added with Kollidon Cl display a packed and dense matrix, bare of pores and completely wrapping the microparticles, addition of the silicate (Neusilin^®^) resulted in a softer and fragmented matrix, in which the polymeric microspheres are still visible as discrete units.

As a consequence, the diffusion of QUE from such systems should follow different pathways and times, as shown in the in vitro release test (Figure 11).

## 3. Materials and Methods

### 3.1. Materials

EUG-B and EUG-C (Evonik Rohm GmbH, Darmstadt, Germany) were kindly provided by Rofarma Italia srl (Gaggiano, Italy). Tween^®^ 80 (polyoxyethylene sorbitan monooleate), Span^®^ 80 (sorbitan monooleate), ethanol and petroleum ether (40–60 °C) were Aldrich products purchased from Merck KGaA (Darmstadt, Germany). Paraffin oil was purchased from Farmalabor Srl (Canosa di Puglia, Italy). Quercetin (QUE) was a Fluka-BioChemika product, purchased from Merck KGaA. The anti-tacking agents were purchased as indicated: talc: Farmalabor (Canosa di Puglia, Italy); Methocel^®^ (methylcellulose): DuPont (Grindsted, Denmark); Mantrocel^®^ K4M (hydroxypropyl methylcellulose): Gustav Parmentier GmbH (Frankfurt am Main, Germany); Aerosil^®^ (silica): Evonik Resource Efficiency GmbH (Hanau-Wolfgang, Germany); Neusilin^®^ UFL2 (synthetic magnesium aluminometasilicate): Fuji Chemical Industries Co., Ltd. (Toyama, Japan); Kollidon^®^ CL (cross-linked poly-vinyl pyrrolidone): BASF Corp. (Cesano Maderno, MB, Italy).

### 3.2. Purification of Eudraguard^®^ Polymers

The commercial EUG-B and EUG-C are made available as 30% (*w/v*) aqueous dispersions containing various amounts of surfactants as emulsifying agents, specifically sodium lauryl sulfate and polysorbate 80 in EUG-B, polyethylene glycol monostearyl ether in EUG-C [17]. A dialysis process was thus developed to remove such unwanted compounds: the polymer suspension was loaded in a semi-permeable membrane (Spectra/Por porous membrane, MWCO: 3.5 KD), closed at the ends with two magnetic clips. The membrane was immersed in a beaker containing 2 L of deionized water and stirred at room temperature for 24 h, during which 3 to 5 changes of the external water were made. At the end the dialyzed mixture was rapidly frozen at −20 °C and then freeze-dried for 24–36 h (Edwards Modulyo, Milan, Italy) to produce a dry white material, which was stored at 4 °C until use.

### 3.3. Preparation of Microparticles

To obtain blank and drug-loaded EUG microparticles, five different preparation techniques, commonly used for microparticle production were tested, applying different formulation variables.

#### 3.3.1. Emulsion-solvent Evaporation Technique (ESE)

An acetone solution (20 mL) containing the polymer and the drug (for the loaded systems) at established concentrations, was dropped by a glass Pasteur pipette into 25 mL of paraffin oil containing 1% (*w/v*) Span^®^ 80, while keeping under magnetic stirring (500 rpm) in an ice bath for about 30 min. The mixture was then left to stir overnight to allow acetone to evaporate.

The following day, petroleum ether was added to the mixture and magnetically stirred for 1 h to disaggregate the microparticles. The mixture was carefully decanted and the collected microparticles were repeatedly washed with petroleum ether and filtered under vacuum using a Hirsch funnel. Finally, the obtained powder was dried under vacuum at 40 °C for 24 h in a Büchi glass oven [31].

Blank and QUE-loaded microparticles were produced using different weight ratios between EUG-B and EUG-C polymers (Table 1).

#### 3.3.2. Solvent Casting Technique (SC)

QUE (10 mg) and polymer (100 mg) were co-dissolved in 2 mL acetone, to achieve a 5% (*w/v*) solution, that was then poured in either a plastic (LD-PE) or silicone mold and allowed to evaporate at room temperature for 2–3 h. The resulting film, having a thickness of about 2 mm, was detached and powdered using a blade mill.

#### 3.3.3. Co-Evaporation Technique (CoE)

The co-evaporation technique involves the preparation of a physical mixture of two or more solid components, dissolved in a common solvent (acetone) and left to stir to allow molecular interaction for about 30 min. The solution is then subjected to the gradual evaporation of the solvent, using a Buchi Rotavapor^®^, in order to obtain a dry material that is recovered and powdered using a ceramic mortar and pestle.

To help the formation of a more homogeneous powder, addition of some pharmaceutical excipients before solvent evaporation was appraised. Talc, methylcellulose (Methocel^®^), hydroxypropyl methylcellulose (Mantrocel^®^), silica (Aerosil^®^), magnesium aluminometasilicate (Neusilin^®^), and cross-linked poly-vinyl pyrrolidone (Kollidon^®^ CL) were thus all tested at various weight ratios with respect to EUG-B polymer (3:1, 1:1, 1:3, or 1:5).

To produce the EUG-B microparticles loaded with QUE at a fixed 1:10 drug-polymer weight ratio, different amounts of anti-tacking agents were tested (Table 5). All the mixtures were dissolved in 20 mL of acetone prior to rotary evaporation.

#### 3.3.4. Quasi-Emulsion Solvent Diffusion Technique (QESD)

An organic phase (2 or 4 mL of a 1% *w/v* solution of the polymer(s) in acetone; cf. (Table 6) was slowly dropped through a thin Teflon tube connected to a syringe into 20 mL of an aqueous solution containing 0.5% (*w/v*) of Tween 80 as a surfactant. The mixture was constantly homogenized at 3000 rpm by an Ultra-Turrax T18 (IKA GmbH, Königswinter, Germany) equipped with a G10 dispersion accessory, for a total time of 15–20 min. Alternatively, a magnetic stirring at 500 rpm and dripping the organic phase using a glass Pasteur pipette for the same total time was investigated.

The interactions between the drug and the organic phase are stronger than the interactions between the organic phase and the aqueous phase, therefore the solvent is dispersed in water producing a ‘quasi-emulsion’ of micro- or nano-droplets. As soon as the emulsion is formed, the solvent and water spread in opposite directions. The water that enters the droplets of organic solution reduces the solubility of the polymer, inducing its crystallization inside the droplets [38].

The mixture was left to evaporate for 24 h under magnetic stirring at room temperature. Microparticles formation occurred through the precipitation of the polymer after the gradual evaporation of the organic solvent and the simultaneous counter-diffusion of the aqueous phase in the dispersed droplets of polymeric solution [39,40].

#### 3.3.5. Solvent Evaporation Technique (SE)

In this procedure, an organic phase consisting of a 1% (*w/v*) polymer (and drug when requested) solution in acetone (6 mL) was slowly dropped (in about 30 min) using a glass Pasteur pipette into 15 mL of an aqueous one consisting of water and Tween 80 at 0.05% (*w/v*), under magnetic stirring (500 rpm) at room temperature. The mixture was left under stirring for 24 h to allow the evaporation of acetone and subsequently frozen at −20 °C and lyophilized for 48 h [14].

### 3.4. Preparation of Physical Mixtures

Physical mixtures (PM) between QUE and EUG-B were prepared under dry conditions by triturating for 30 min in a porcelain mortar the two ingredients in a 1:5, 1:10 and 1:25 drug-to-polymer weight ratio.

### 3.5. Microparticle Characterization

Selected preparations subjected to solid-state physico-chemical characterization by Scanning Electron Microscopy (SEM), FT-IR spectroscopy, differential scanning calorimetry (DSC) and powder X-ray diffractometry (PXRD) analysis.

The morphology of microparticles was studied using a SEM EVO 15 (Zeiss, Cambridge, UK). Each sample was positioned on a sample stub and was gold sputtered with a thin layer (≈10 nm) of gold before the SEM analysis without any other pre-treatment being applied. The SEM analysis was carried out with 15 keV voltage and the magnification varied from 100× to 2000× for sample ESE.B.Q5, from 100× to 2500× for sample ESE.B.Q10, and from 100× to 1500× for sample ESE.B.Q25.

IR analysis was performed using a Fourier transform instrument (1600 FT-IR spectrophotometer; Perkin Elmer Italia SpA, Milan, Italy). Dry microparticles were mixed with KBr to produce transparent wafers. The spectrum was recorded in the range 4000–500 cm^−1^. For DSC studies, a DSC 1 STAR SYSTEM calorimeter (Mettler-Toledo Spa, Novate M., Italy) was used, connected to a Haake D8-G thermostat (Thermo Fisher Scientific, Waltham, MA USA 02451).

Temperature and enthalpy variations were calibrated using a pure indium sample. The detection system consists of a Mettler Pt100 sensor, with a thermodynamic sensitivity of 56 nV/°C, a calorimetric sensitivity of about 3 nV/mW and a background noise of 60 nV (<1 mV). Each DSC scan had an accuracy of ±0.4 °C and a reproducibility and resolution of 0.1 °C. To determine the thermotropic behavior, each sample (10 mg) was sealed in a 100 µL aluminum pan; an empty pan was used as a reference. Samples were submitted to a heating cycle between 20 and 330 °C, at a scan rate of 5 °C/min.

PXRD data were recorded with an X’Pert Pro X-ray diffraction system (Philips, Eindhoven, The Netherlands) (available at the Centro Interdipartimentale Grandi Strumenti di Modena e Reggio Emilia — CIGS) equipped with a PAN analytical solid state detector, operating in reflection mode, with a CuKα radiation (without monochromator: λ is a “mixing” between λKα1 = 1.540598 and λKα2 = 1.544426; Kβ radiation was removed by a Ni foil). X-ray data were collected over a range 5 < 2θ < 30 at room temperature; the scan-rate was set at 0.007°/s.

To evaluate the drug content and drug encapsulation efficiency, microparticle specimens were weighed and dissolved in methanol (approximately in a 1:10 *w/v* ratio) under magnetic stirring for 30 min. The solutions were analyzed by an UV spectrophotometer (Genesis 10w Scanning ThermoElectron, Waltham, MA, USA), at a wavelength of 377 nm. QUE concentration was obtained against a calibration curve of the drug in methanol (linear in the range 0–50 µg/mL; r^2^ = 0.9999). 

Drug content (DC%) was calculated according to the following formula:

(1) $$DC\%=\frac{Actual\;drug\;amount}{produced\;microparticles\;amount}\times 100;$$

Encapsulation efficiency (EE%) was calculated according to the formula:

(2) $$EE\%=\frac{Actual\;drug\;amount}{theoretical\;drug\;amount}\times 100.$$

### 3.6. QUE Dissolution Assay

To determine the dissolution rate of the neat drug, an ERWEKA: DZT Dissolution paddle tester for solid pharmaceutical forms was used (ERWEKA GmbH, Langen, Germany), reproducing in vitro the physiological conditions of the gastrointestinal tract. The test was carried out at 100 rpm and 37 ± 0.5 °C. First, the amount of drug to be used in each experiment was calculated in order to respect the sink conditions. Drug samples were poured into a gelatin capsule (no. 1) and soaked in the suitable dissolution medium. The three different environments of the GI tract were reproduced following the corresponding pH conditions using simulated gastric fluid (SGF, pH 1.2) for 2 h, then simulated intestinal fluid (SIF) (pH 6.8) for 4 h and finally a phosphate buffered solution at pH 7.4 up to 24 h. To simulate the acidic environment of the stomach, a solution of 0.1 N HCl (187.5 mL) was inserted in the dissolver beaker (SGF, pH 1.2). After 2 h, 62.5 mL of a 0.2 M tribasic sodium phosphate solution, pre-heated at 37 ° C, were added, thus raising the pH to 6.8 (intestinal value). After further 4 h, the pH was raised to 7.4 with a few drops of 2 N sodium hydroxide solution.

During the 24 h of the assay 2-mL aliquots were periodically withdrawn and the test vessel was added with the same volume of the pertinent dissolution medium, to restore the original volume. Taken samples were centrifuged at 10,000 rpm and 10 °C for 30 min and the clear supernatant was analyzed by UV spectrophotometry; absorbance was correlated with a calibration curve of QUE in the two reference solutions (0.1 N HCl and pH 6.8 phosphate buffer), both linear in the range 0–20 µg/mL (r^2^ = 0.9998), calculating the respective concentrations. The experiment was repeated in triplicate.

### 3.7. In Vitro Release Studies

The loaded formulations obtained from the preparation methods Co and ESE were subjected to pH-dependent release tests, according to the method described for the dissolution assay. Each sample was tested in duplicate or triplicate.

Two-mL aliquots were withdrawn at established intervals and replaced with the same volume of 0.1 N HCl or phosphate buffer solution (at 6.8 and 7.4, respectively). The collected samples were centrifuged at 10,000 rpm and 10 °C for 30 min and the clear supernatant was analyzed by UV spectrophotometer to calculate the drug concentration.

## 4. Conclusions

This work was aimed at the optimization of the process of production of specific polymeric preparations for colonic drug delivery using the Eudraguard^®^ Biotic and Control methacrylate copolymers, and evaluating different formulation approaches.

The QESD and solvent evaporation (SE) techniques have highlighted formulation problems consisting in the formation of filamentous aggregates and rubbery clusters consequent to the chemical-physical properties of the copolymers. Both techniques are characterized by the use of an aqueous dispersing phase in which the organic phase is dropped, under magnetic stirring for the SE method or high-speed homogenization using an UltraTurrax apparatus for the QESD method.

To overcome this problem, formulation variations, including changes in the volumetric ratios of the two preparation phases and mother solutions at different concentrations, were tested. These variations highlighted the formation of aggregates already during the drip phase for mother solutions with concentrations greater than 1% *w/v* in copolymer during the evaporation phase of the acetone for volumetric ratios between the two phases lower than 1:10.

The formation of these aggregates/clusters would seem to be ascribed to the high difference in solubility of the copolymers between the two preparation phases resulting in rapid co-crystallization at the interface. The inability to continue effectively with the freeze-drying phase and the final volume led to the choice of anhydrous techniques.

Thin films of difficult recovery and shredding have been obtained through the evaporation of acetone from the polymer and drug solutions (CoE technique). A series of excipients used for tablet production were added to facilitate to obtain homogeneous powders and to overcome the problem of recovery/collection. This strategy led to the formation of poorly porous systems unable to warrant a satisfactory release of the encapsulated drug.

Conversely, through the ESE technique satisfactory dry powders were obtained; in particular, the morphological analysis of microparticles showed that a minimal drug-polymer weight ratio above 1:10 was necessary to achieve a complete and homogenous encapsulation of the active ingredient within the EUG network. By the SC method, in which the organic polymer solution was poured into a plastic or silicone mold, thin films were obtained that could be easily converted in dry powders. Both these last techniques allowed to create the best systems in terms of gross appearance, morphology and work-out prospect. The in vitro release studies have shown an inhibition of the release at the gastric level and a good specific colon release, particularly with the last technique and when the ratio of copolymer was sufficient to ensure a complete encapsulation of the drug.

Based on these preliminary interesting findings, further studies aimed at evaluating other aspects, such as the determination of the correct weight ratios between these two copolymers able to ensure an efficient drug release control, are enviable to better exploit the application of Eudraguard^®^ polymers in the controlled delivery of nutraceutical active compounds.

Also, the possibility of mixing such materials with other food-grade polymers would be interesting to be evaluated, in order to obtain a better control of the drug release and more stable and industrially scalable systems.

In addition, systems with different model drugs (belonging to different classes of solubility) have been already planned, to identify the interaction characteristics and determine the effectiveness of the system release.

## Figures and Tables

**Table 1:** Composition of blank and QUE-loaded microparticles produced by the ESE technique.

Batch	Polymer	Polymer(s) (mg)	QUE (mg)	Drug: Polymer Weight Ratio
ESE.B.0	EUG-B	250	-	-
ESE.C.0	EUG-C	250	-	-
ESE.BC.0	1:1 EUG-B + EUG-C	250	-	-
ESE.B.Q10	EUG-B	250	25	1:10
ESE.C.Q10	EUG-C	250	25	1:10
ESE.BC.Q10	1:1 EUG-B + EUG-C	250	25	1:10
ESE.B.Q5	EUG-B	250	50	1:5
ESE.B.Q25	EUG-B	250	10	1:25

**Table 2:** IR analysis (KBr, cm^−1^) of QUE and its 1:10 physical mixture (PM) and microparticles with EUG-B and EUG-C (cf. Table 1 for batch composition).

Attribution	QUE ([32])	QUE (exp.)	(Dried) EUG-B	(Dried) EUG-C	QUE−EUG-B 1:10 PM	ESE.B.Q10	ESE.C.Q10	ESE.BC.Q10
OH stretching	3406, 3283	3408	3400–2400	-	3409	3447	3421	3447
C–H stretching	-	-	2956–2980	2925, 2820	-	2940	2984, 2960	2890
C=O stretching	-	-	1734	1734	-	1734, 1710	1734	1750
Aryl ketone C=O stretching	1666	1665	-	-	1664	1654	1654	1684
Aromatic ring C=C stretching	1610, 1560, 1510	1613, 1560, 1522	-	-	1612, 1560, 1522	1617, 1560, 1508	1616, 1560, 1522	1654–1522
CH_2_ bending	-	-	1458	1508	1449	1458	1458	1458
CH_3_ bending	-	-	1388	1360, 1310	1383	1388	1386	1390
Phenol OH bending	1379	1383	-	-	1383	1380	-	1387
In-plane bending of aromatic C–H	1317	1320	-	-	1320	1325	1312	-
C–O stretching (aryl ether ring)	1263	1263	-	-	1263	1255	1265	1267
Phenol C–O stretching	1200	1199	-	-	1200	1195	-	1190
Ketone C–CO–C stretching and bending	1165	1169	-	-	1169	1168	1169	1160
C–O stretching	-	-	1163–1250	1170	1169	-	-	1168
Out-of-plane bending	930, 820, 679, 600	942, 825, 681, 604	972	-	930, 824, 681, 604	950, 827, 670, 656	851, 824, 670, 601	950–900, 826

**Table 3:** DC and EE values of QUE-EUG microparticles produced by the CoE technique.

Batch	QUE: Polymer Weight Ratio	%DC	%EE
A1Q10	1:10	3.55 ± 0.33	39.05 ± 3.63
H1Q10	1:10	4.36 ± 0.23	48.29 ± 2.51
N1Q10	1:10	4.06 ± 0.09	44.66 ± 0.99
K1Q10	1:10	3.34 ± 0.10	36.74 ± 1.10
K2Q10	1:10	4.60 ± 0.28	50.60 ± 3.07

**Table 4:** DC and EE values of QUE-EUG microparticles produced by the ESE technique.

Batch	QUE: Polymer Weight Ratio	% DC	%EE
ESE.B.Q10	1:10	8.78 ± 1.00	97.6 ± 11.11
ESE.C.Q10	1:10	6.14 ± 0.33	68.9 ± 3.67
ESE.BC.Q10	1:10	7.92 ± 0.91	87.9 ± 10.11
ESE.B.Q5	1:5	15.28 ± 0.31	92.8 ± 3.44
ESE.B.Q25	1:25	3.68 ± 0.07	96.9 ± 7.76

**Table 5:** Composition of QUE-loaded EUG-B microparticles produced by the CoE technique.

Batch	Excipient	Polymer: Excipient Weight Ratio
A1Q10	Aerosil	1:3
H1Q10	Mantrocel K4M	1:3
N1Q10	Neusilin	1:3
K1Q10	Kollidon CL	1:3
K2Q10	Kollidon CL	1:1
K3Q10	Kollidon CL	2:1
K4Q10	Kollidon CL	3:1

**Table 6:** Composition of blank microparticles produced by the QESD technique.

Batch	Polymer	Volume (mL) of 1% (*w/v*) Polymer Solution Added to 20 mL of the Aqueous Phase
EUB.D.0	EUG-B	2
EUB.D2.0	EUG-B	4
EUC.D.0	EUG-C	2
EUBC.D.0	1:1, EUG-B + EUG-C	2

## Supplementary Materials

The following are available online at https://www.mdpi.com/1424-8247/13/6/131/s1, Figure S1: IR spectrum of pure quercetin; Figure S2: IR spectrum of dried Eudraguard Biotic (EUG-B); Figure S3: IR spectrum of commercial Eudraguard Control (EUG-C) (source: Evonik website); Figure S4: IR spectrum of blank ESE microparticles made by 100% Eudraguard Control; Figure S5: IR spectrum of blank ESE microparticles made by 50% Eudraguard Control and 50% Eudraguard Biotic; Figure S6: IR spectrum of Eudraguard Biotic ESE microparticles loaded with quercetin (1:10 drug-to-polymer ratio); Figure S7: IR spectrum of Eudraguard Control ESE microparticles loaded with quercetin (1:10 drug-to-polymer ratio); Figure S8: IR spectrum of Eudraguard Biotic/Control (1:1, w/w) ESE microparticles loaded with quercetin (1:10 drug-to-polymer ratio); Figure S9: IR spectrum of an 1:10 quercetin-Eudraguard Biotic physical mixture.
