# Supplementary material for: Development, Optimization and Characterization of Eudraguard®-Based Microparticles for Colon Delivery
Source: Pharmaceuticals (Basel). 2020 Jun 24;13(6):131. doi: 10.3390/ph13060131 (PMC7344638; doi:10.3390/ph13060131)
Supplement: Supplementary file 1 [file pharmaceuticals-13-00131-s001.pdf]

Development, optimization and characterization of Eudraguard®-based microparticles for colon delivery

Claudia Curcio[1], Antonio S. Greco[1], Teresa Musumeci[1], Salvatore Rizzo[1], Barbara Ruozi[2],

Rosario Pignatello[1]*

SUPPLEMENTARY MATERIAL: IR SPECTRA OF QUERCETIN, EUDRAGUARD POLYMERS AND THE RELATIVE MICROPARTICLES AND PHYSICAL MIXTURE.

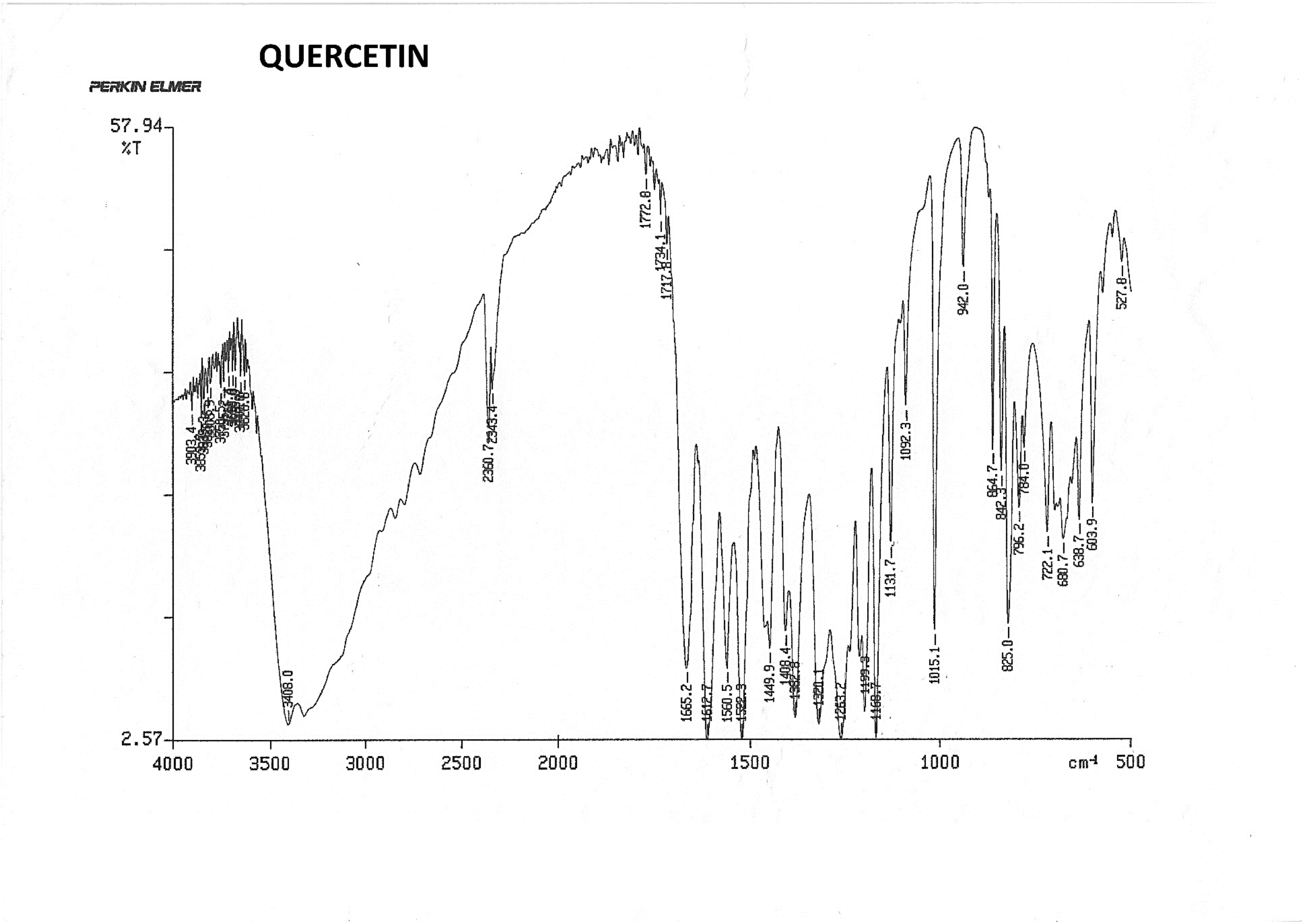

Figure S1. IR SPECTRUM OF PURE QUERCETIN

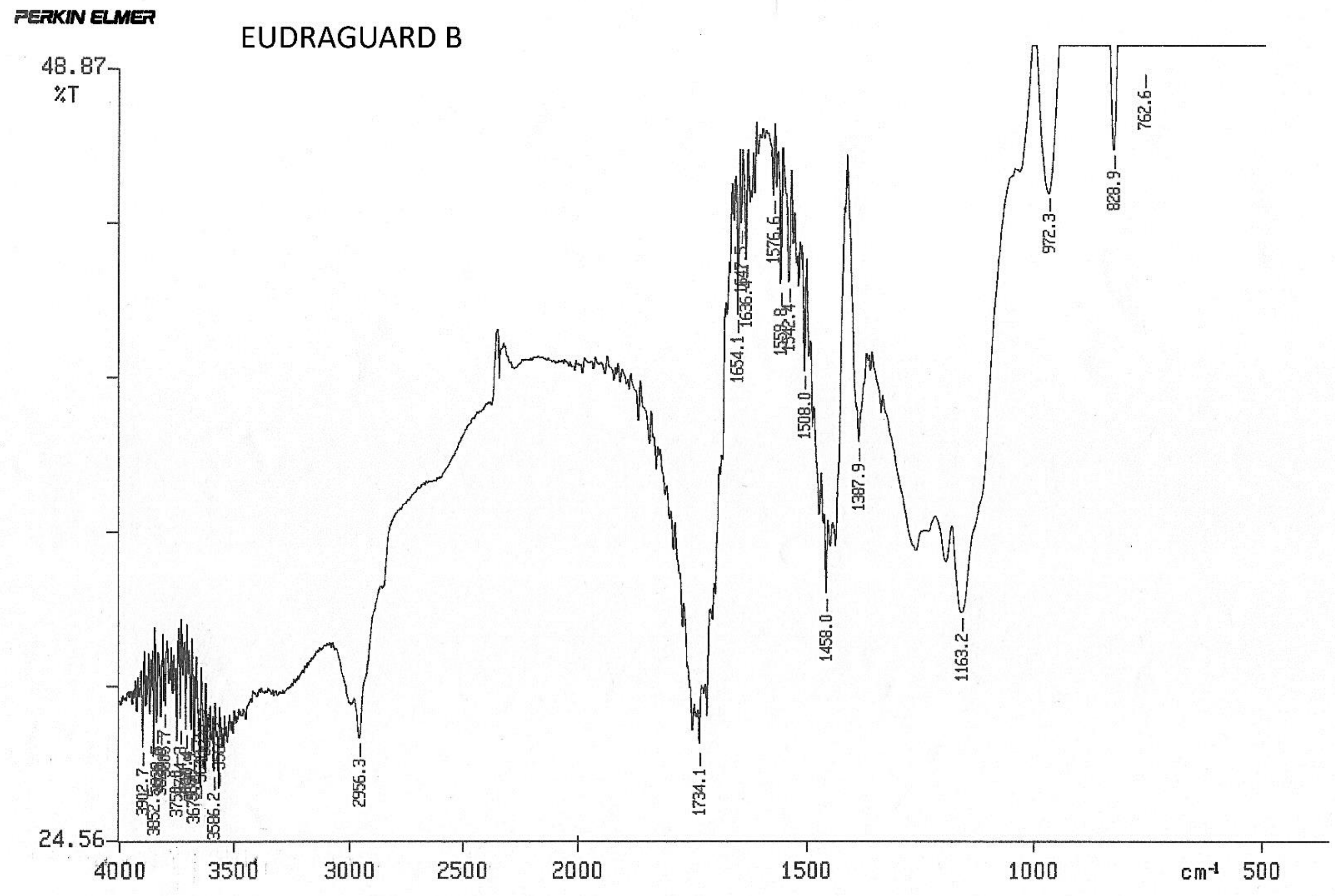

Figure S2. IR SPECTRUM OF DRIED EUDRAGUARD BIOTIC (EUG-B)

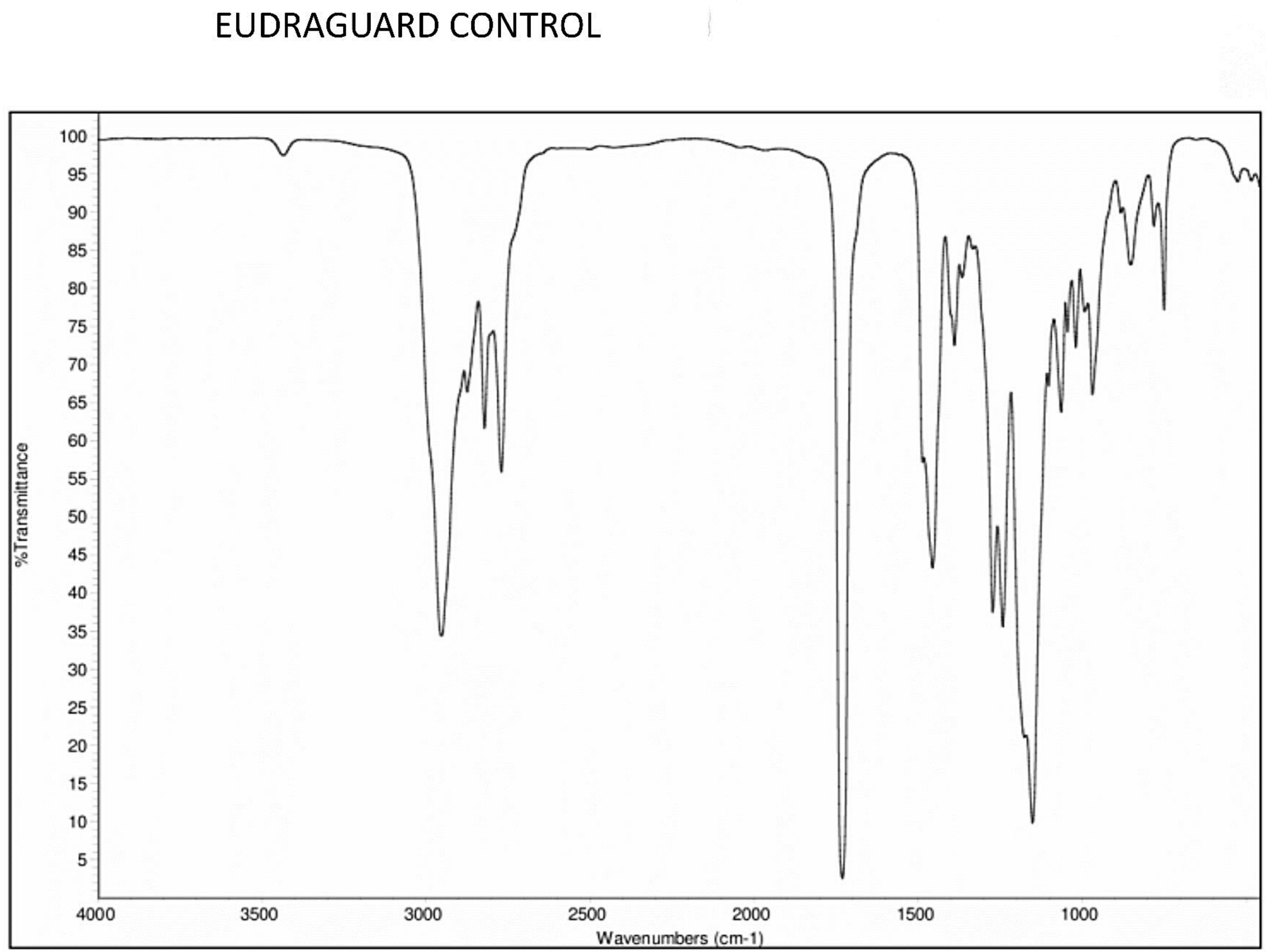

Figure S3. IR SPECTRUM OF COMMERCIAL EUDRAGUARD CONTROL (EUG-C) (source: Evonik website)

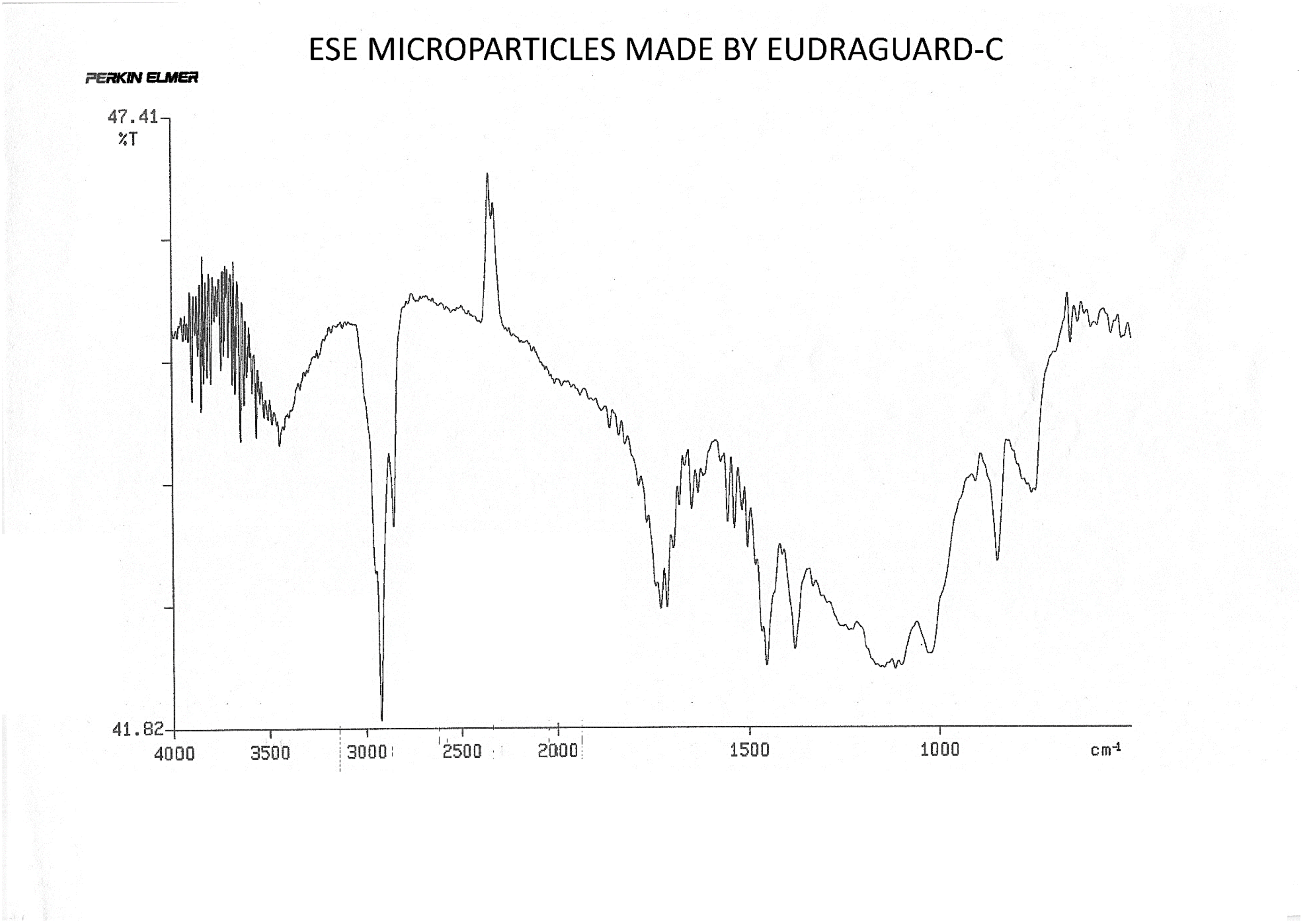

Figure S4. IR SPECTRUM OF BLANK ESE MICROPARTICLES MADE BY 100% EUDRAGUARD CONTROL

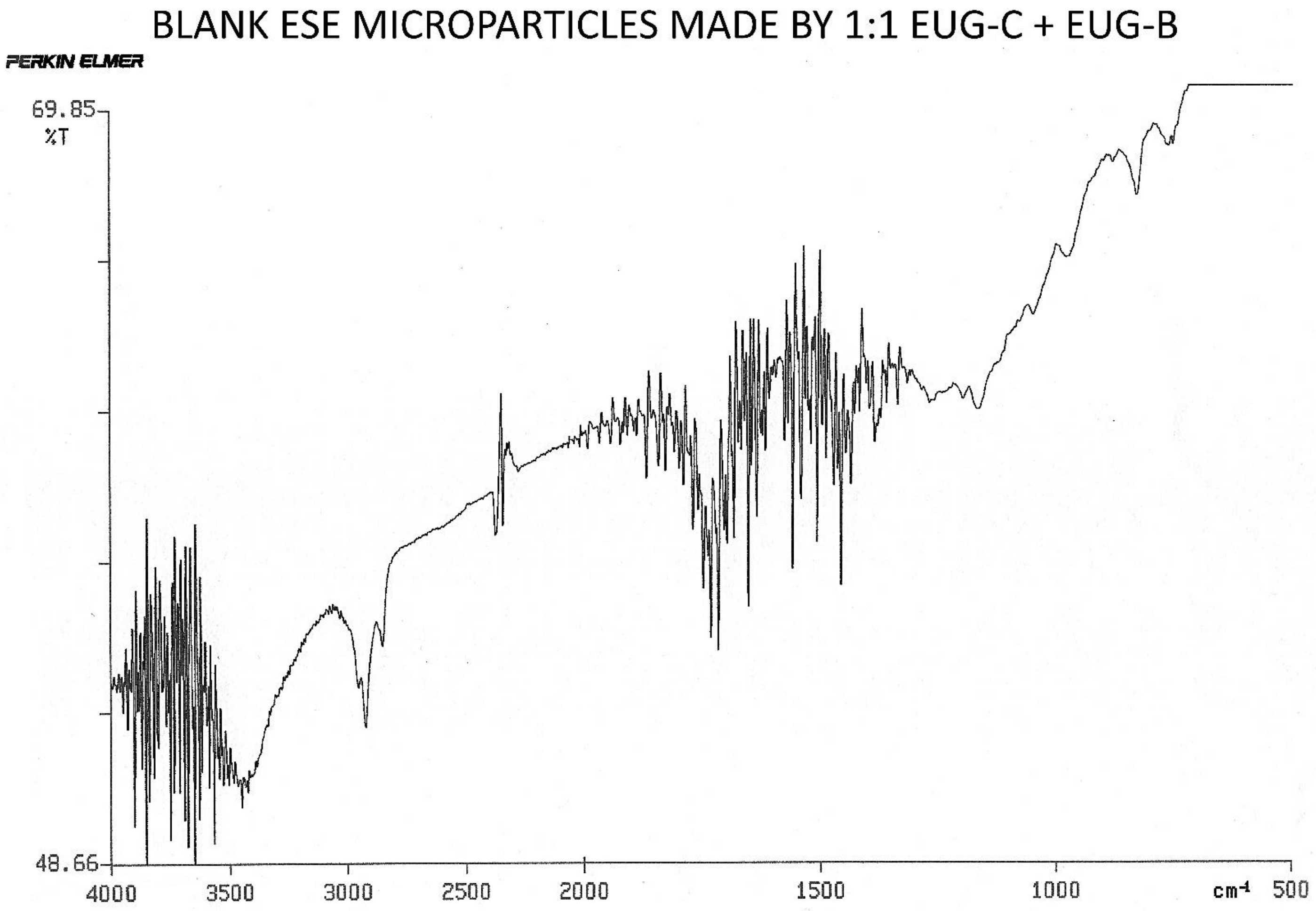

Figure S5. IR SPECTRUM OF BLANK ESE MICROPARTICLES MADE BY 50% EUDRAGUARD CONTROL AND 50% EUDRAGUARD BIOTIC

EUDRAGUARD-B ESE MICROPARTICLES LOADED WITH QUERCETIN (10:1, w/w)

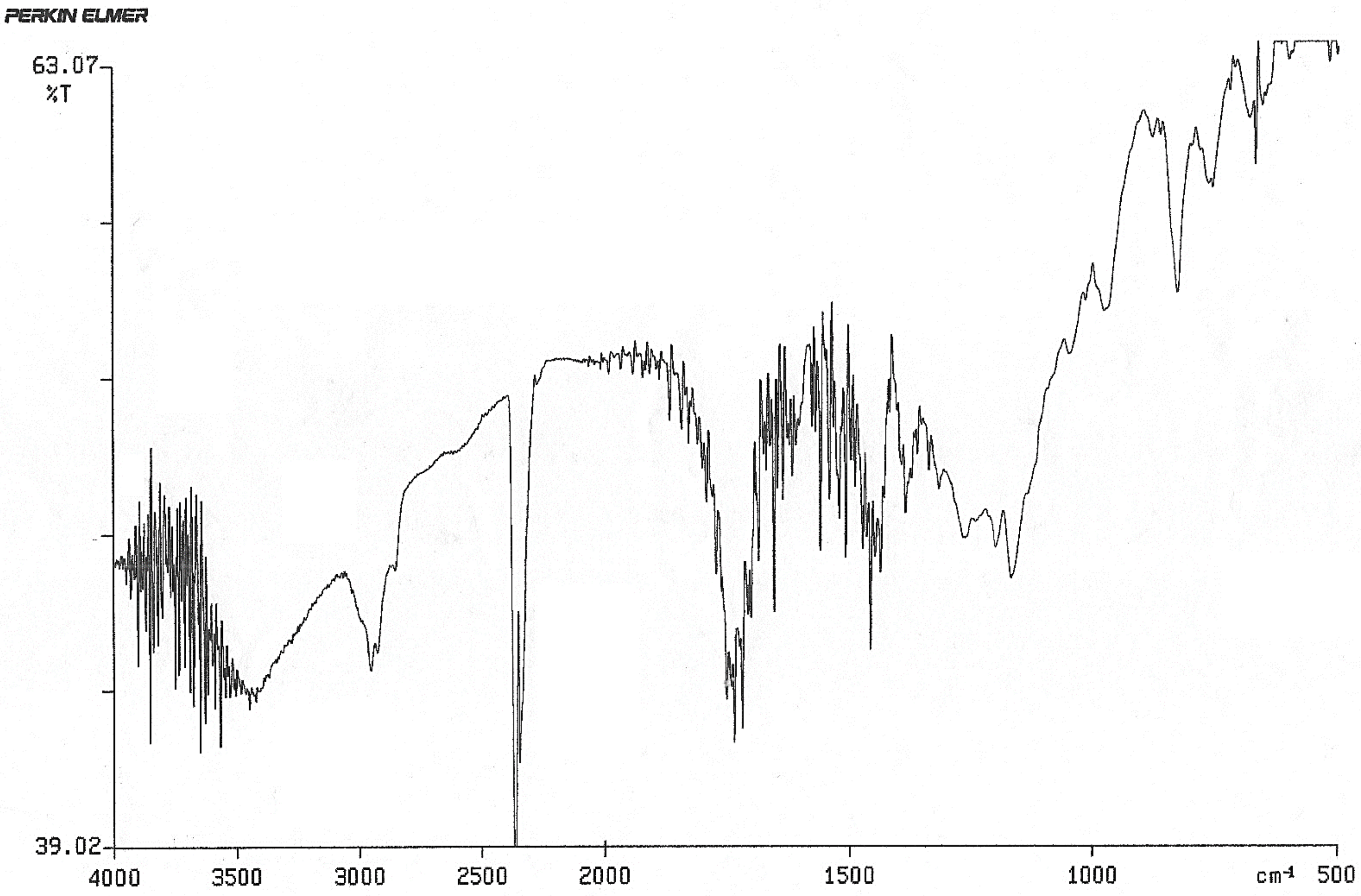

Figure S6. IR SPECTRUM OF EUDRAGUARD BIOTIC ESE MICROPARTICLES LOADED WITH QUERCETIN (1:10 DRUG-TO-POLYMER RATIO)

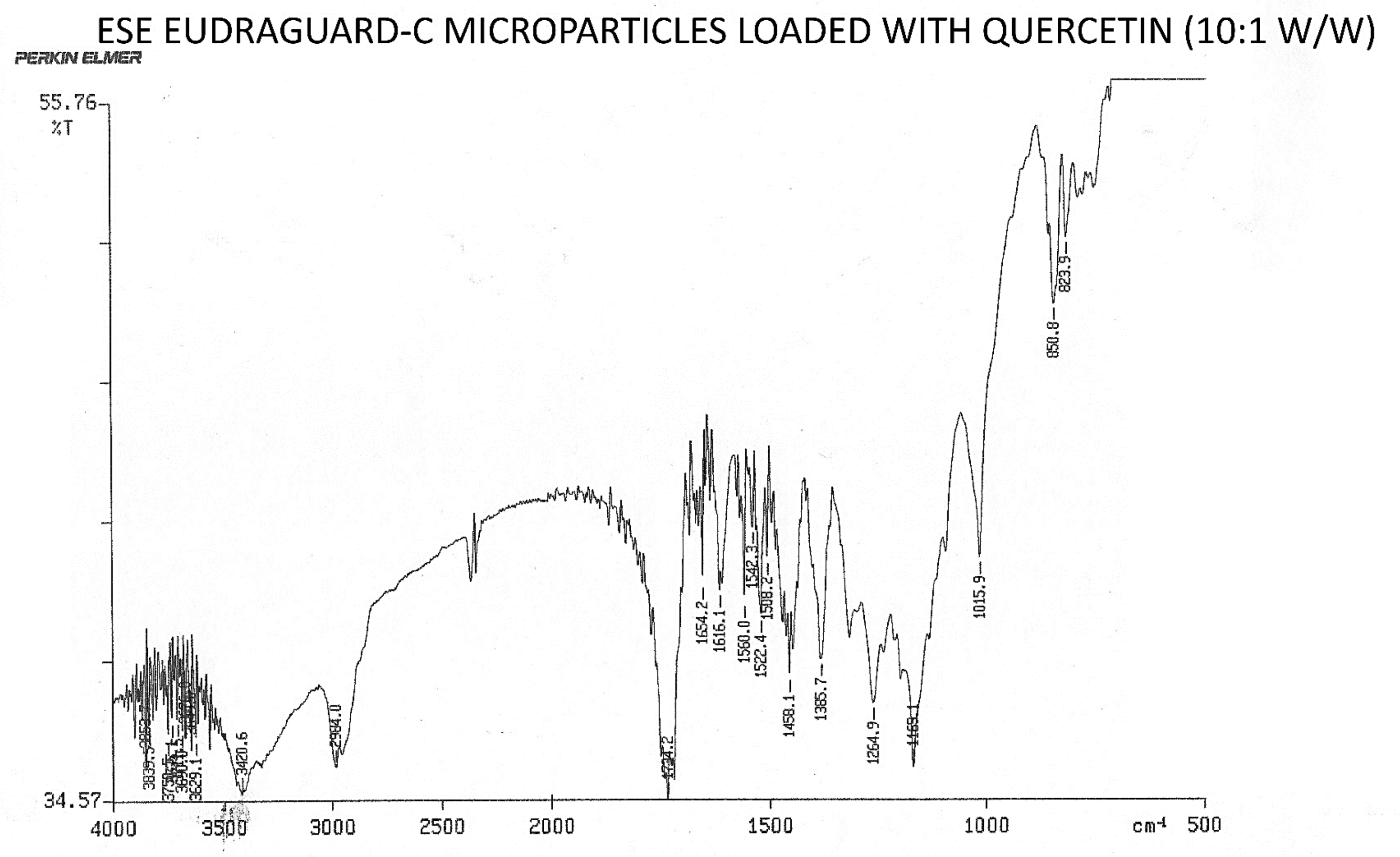

Figure S7. IR SPECTRUM OF EUDRAGUARD CONTROL ESE MICROPARTICLES LOADED WITH QUERCETIN (1:10 DRUG-TO-POLYMER RATIO)

EUDRAGUARD B+C (1:1) ESE MICROPARTICLES LOADED (10:1, w/w) WITH QUERCETIN

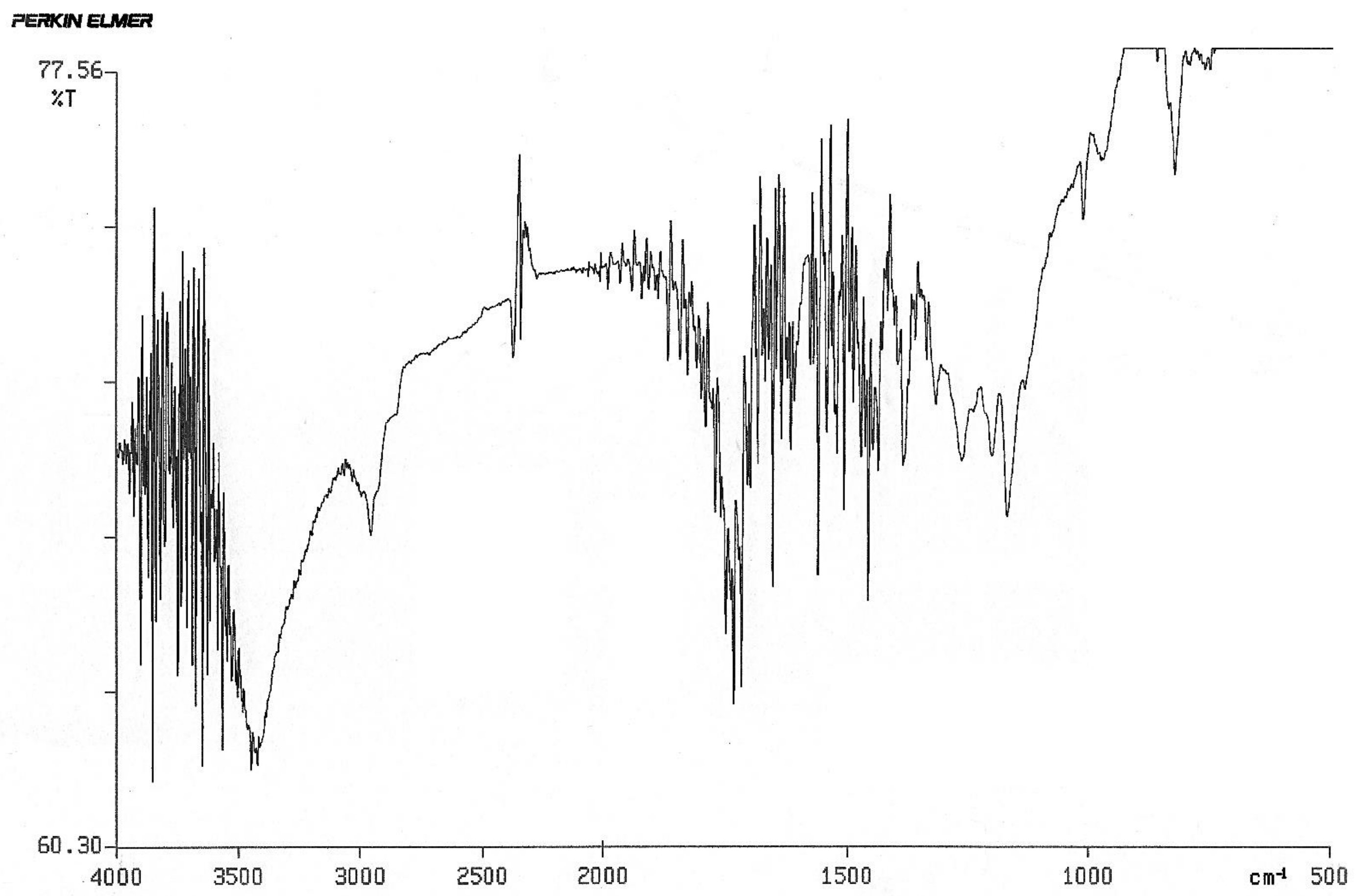

Figure S8 . IR SPECTRUM OF EUDRAGUARD BIOTIC/CONTROL (1:1, W/W) ESE MICROPARTICLES LOADED WITH QUERCETIN (1:10 DRUG-TO-POLYMER RATIO).

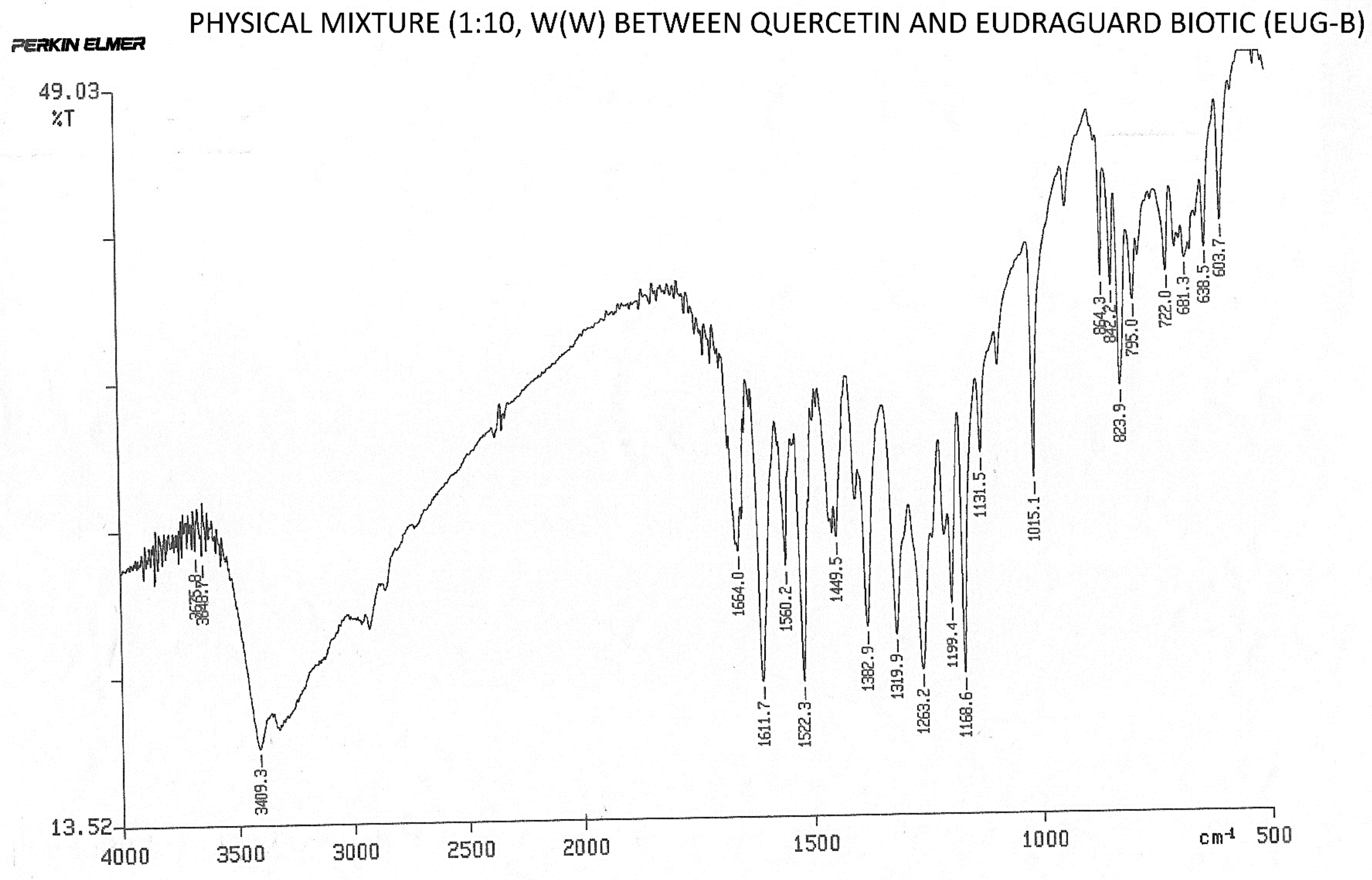

Figure S9. IR SPECTRUM OF AN 1:10 QUERCETIN-EUDRAGUARD BIOTIC PHYSICAL MIXTURE
